# Some experiments and remarks regarding the possible formation of blood stains on the *Turin Shroud*: stains attributed to the crown of thorns, the lance wound and the belt of blood

**DOI:** 10.1007/s00414-023-02959-6

**Published:** 2023-02-11

**Authors:** Lisa König, Ronja Schmölders, Marcel Jühling, Alexandra Reckert, Anna Heger, Stefanie Ritz-Timme

**Affiliations:** Institute of Legal Medicine, University Hospital Düsseldorf, 40225 Düsseldorf, Germany

**Keywords:** Turin Shroud, Crown of thorns, Lance wound, Belt of blood

## Abstract

The *Turin Shroud* (TS) is a Christian relic interpreted to be the burial cloth of Jesus of Nazareth. It exhibits red discolorations that have been interpreted as blood stains and that are the subjects of a highly controversial discussion. We conducted experiments to identify theoretically possible explanations for the stains attributed to the *crown of thorns*, the *lance wound* and the *belt of blood*. In the experiments with a focus on the stains attributed to the *crown of thorns*, a very similar stain pattern as on the TS could be provoked by simulating the following sequence of events: blood from antemortem scalp wounds is covering hair and face; blood is coagulating and/or drying; blood components are mobilised by postmortem washing and oiling. A stain pattern very similar to the *belt of blood* on the TS was successfully provoked by simulating the following sequence of events: The body is lying in a supine position, blood or bloodied water flowing from a wound at the right lateral chest wall; the body is rotated to the left side; the Shroud is tucked under the back; the body is rotated back to a supine position and laid onto the Shroud. The so-called *serum ring* surrounding the stain attributed to the *lance wound* could be reproduced by sequential application of serum and whole blood samples or of pleural effusion and whole blood samples onto cotton cloth. It is obvious that any attempt to interpret the assumed blood stain pattern on the TS has serious limitations. Nevertheless, it seems remarkable that we were able to reproduce findings that appear to be very similar to stains on the TS.

## Introduction

**Fig. 1:**
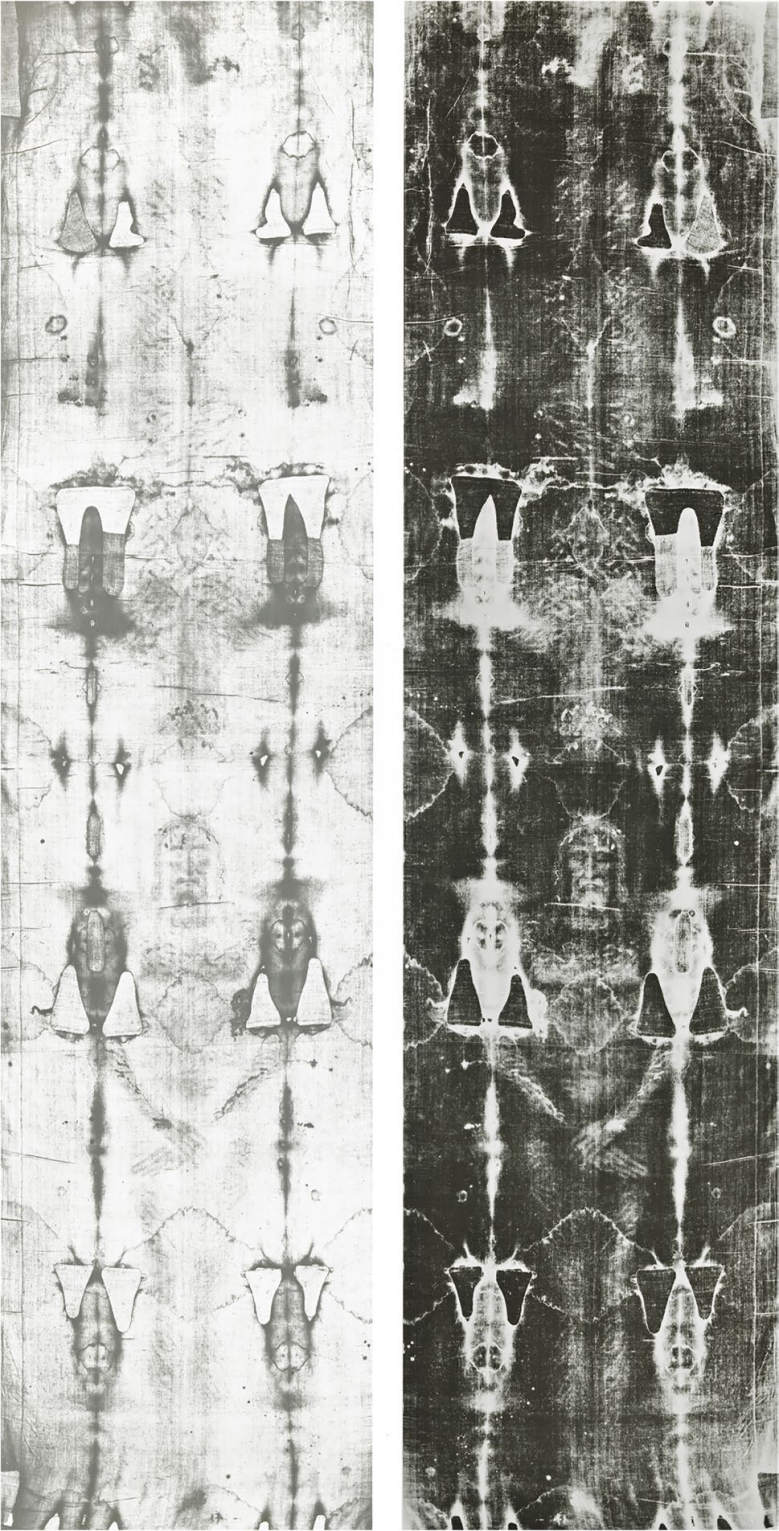
Positive (left) and negative (right) photographs of the *Turin Shroud* (TS), depicting the frontal and dorsal image of a male human body (*photo from *www.shroudphotos.com*, ©Vernon Miller, 1978*)

The *Turin Shroud* (TS) is a linen cloth (4.4-m long, 1.1-m wide) depicting the frontal and dorsal image of an obviously male human body (Fig. 1), hereafter referred to as *Turin Shroud Man* (TSM). As a Christian relic, the Shroud is interpreted to be the burial cloth of *Jesus of Nazareth* and is fascinating to and worshipped by many Christian believers.

At the same time, the TS is a highly controversial subject of a huge amount of studies published in peer-reviewed and (frequently) in non-peer-reviewed journals and other media. A *Google Scholar* search of the keyword *Turin Shroud* revealed 1070 papers published since 2021 (search in December 2022). It is remarkable that there are so many studies despite the limited access to the TS. Until now, all research is based on some physical samples taken before 1988 [1] and on photographs taken in 1898, 1931, 1969 and 2002 [1], not all of which are publicly available.

The studies address many questions (for a review see [1, 2]), e.g. the characteristics of the cloth, the dating of the relic, the characteristics and the formation of the body image as well as (forensic) medical issues like blood and body fluids on the linen and the cause of death of the TSM [3, 4].

A lecture request drew our attention to the possible blood stain pattern on the TS. On the linen, areas with red discoloration are depicted that have been interpreted as blood stains from injuries caused by torture and crucifixion and are therefore viewed as an indication for the authenticity of the TS being the burial cloth of the crucified Jesus Christ [2]. Although older studies have assumed that the red discolorations are due to traces of blood [1, 2], this has not yet been proven by modern scientific methods [4]. However, for the purpose of this paper, the designation of the red discolorations being blood stains will not be challenged.

Numerous hypotheses regarding the origin of the blood stains and the circumstances and cause of death of the TSM have been presented in the literature. We noticed that some hypotheses—from a forensic scientist’s viewpoint—do not seem to be scientifically well-founded and partly even contradict common forensic medical experience. In particular, this concerns some general assumptions about the origin of the blood stains as well as the interpretation of some specific complexes of blood stains, such as those due to injuries caused by the *crown of thorns* and the *lance wound*.

### General assumptions about the origin of the blood stains

Whereas the image of the body itself is reminiscent of a photographic negative, the blood stains appear as positive imprints on the cloth; this finding has been interpreted as an indication that the body image and the blood stains are the results of differing mechanisms of formation [4, 5]. Mostly, the blood stains seem to overlay the body image, and some appear outside the perimeters of the body image [6]. According to Bucklin [6], the stains appear carmine coloured on colour photos and “internal structures […] with physical separation of red blood cells from serum and localisation of the cells toward the periphery of the blood deposits” were observed on “enhancement photos made with Digital Image Analysis and Display System”. Jumper et al. [7] emphasise that “there is evidence of some apparently colourless fluid bordering or diffusing farther out than the darker fluid”.

By some authors, the origin of the blood stains has been attributed to an “external fibrinolytic” (“melting”) activity that enabled a postmortem transfer of (primarily coagulated) blood from the body to the linen [4, 8–10] and by other authors to a postmortem smudging of blood clots in a humid environment [4, 6, 11].

Lavoie et al. [11] differentiated between blood stains from “clots” (at the head and the arms) and those from the “scourge marks and the blood flow on the dorsal image” as a “different category regarding their formation and moisture content”. The group performed experiments to investigate the feasibility of a blood transfer from clots on cling film or skin to linen. In their experiments, a “fairly good mirror image” could be transferred from cling film to linen within 1.5 h (without moistening of the clots) and within 2.5 h after moistening the clots with saline solution 15 min before sampling; moistened clots from the skin had to be transferred to the linen within 1 h for a good result. The authors emphasise that blood from a clot can only be transferred if the clot is moist, and that “in this state, the clot can be very easily disturbed”. Since “the clot images seen on the Shroud” were “intact and well-defined”, the authors concluded that “extreme care had to be taken in the removal of the body from the vertical position to its subsequent horizontal placement in the Shroud”.

For the interpretation of the blood stains on the TS, it is relevant to consider whether the TSM was washed prior to being wrapped in the Shroud. The controversial discussion regarding this question “has far reaching significance in terms of [the matter of] authenticity of the Shroud of Turin” [12]. Referring to the literature, the Bible and rabbinical works, Lavoie et al. [13] argued that the body of Jesus was not washed because—according to Jewish burial customs—a body was not washed if “death is by violence and blood flows at the time of death”. According to Zugibe [12], however, this assumption is incompatible with the blood stain pattern on the TS. In his opinion, the preciseness and the exact, undisturbed pattern of blood stains can only be explained and are only plausible if the body had been washed before being placed on the burial cloth. The author bases his statement on a discussion of Jewish burial customs and on an analysis from the perspective of a forensic pathologist as well as on postmortem washing experiments. After postmortem washing of antemortem-inflicted wounds of an accident victim, “reasonable good impressions of the wounds” could be produced on linen and paper towels that were gently touched against the wounds; in another experiment, small rivulets of “blood” appeared after wounds were briefly washed postmortem.

### Blood stains attributed to injuries caused by the *crown of thorns*

**Fig. 2:**
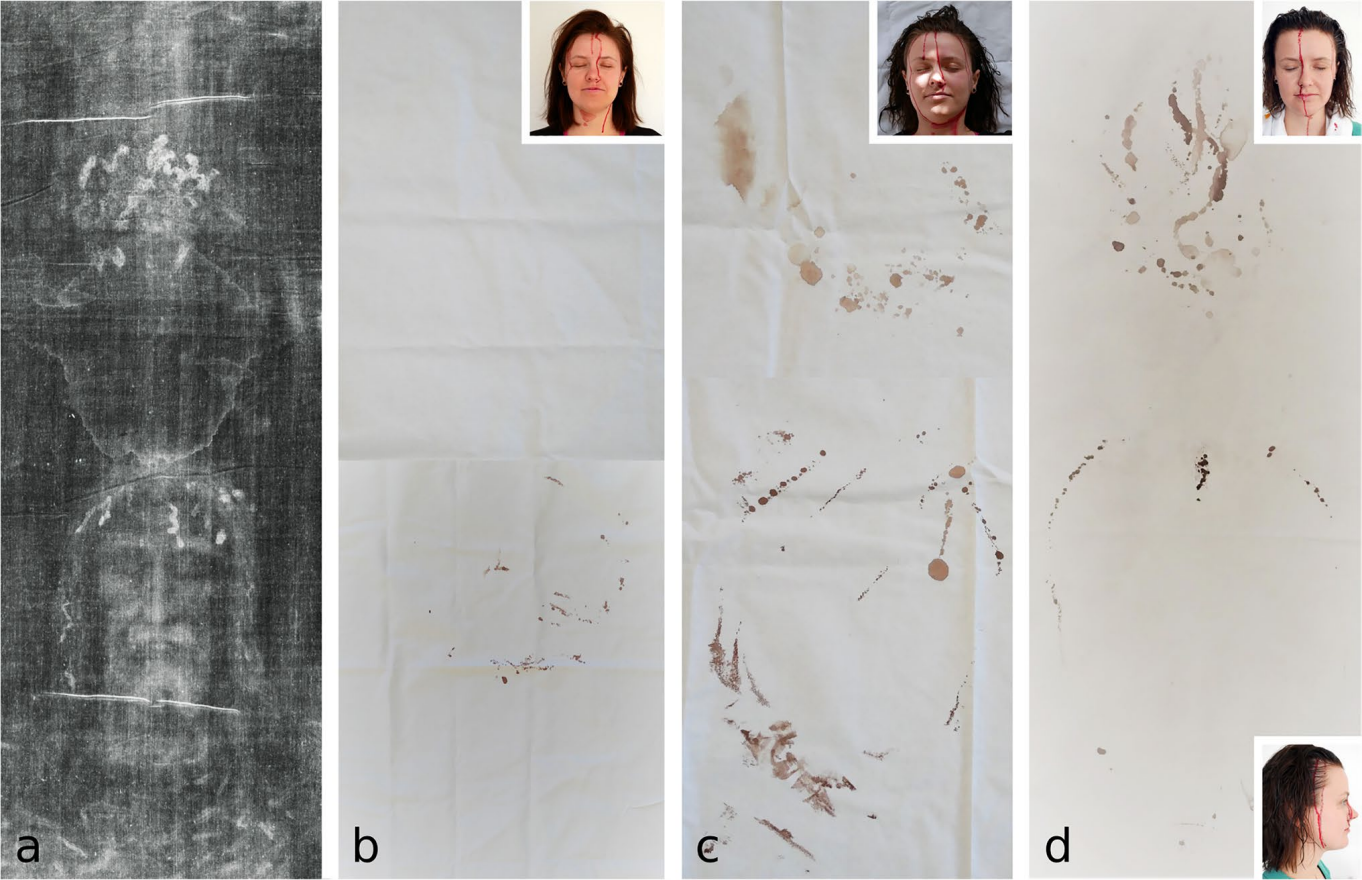
Experimental findings regarding the complex “blood stains attributed to injuries caused by the *crown of thorns*”, in comparison with the corresponding “blood stains” on **a** the *Turin Shroud* (TS) (*photo from *www.shroudphotos.com*, ©Vernon Miller, 1978*); **b** simulation 1a: *liquid/moist blood from antemortem scalp wounds is covering the hair and the face*; **c** simulation 1b: *blood from antemortem scalp wounds is covering the hair and the face, blood is coagulating and/or drying and blood components are mobilised by postmortem washing*; (**d**) simulation 2: as simulation 1b but with additional oiling of face and hairline. The findings in **d** seem to be most similar to the findings on the TS (**a**)

Svensson and Heimburger [4] described the blood stains on the TS “as in an autopsy room”. The blood stains in the area of the head (Fig. 2a) were described by the authors as “meandering rivulets running downwards by gravity in the hair, the forehead and the back of the head” and a “characteristic meandering, broadening figure ‘3’ rivulet on the forehead” was highlighted [4].

With regard to the Gospels of John (“And the soldiers platted a crown of thorns, and put it on his head […]”, John 19:2 [14]), Matthew (“And when they had platted a crown of thorns, they put it upon his head […]”, Matthew 27:29 [14]) and Mark (“And they […] platted a crown of thorns, and put it about his head”, Mark 15:17 [14]), these blood stains were predominantly assigned to injuries caused by the *crown of thorns* [4], possibly a “cap-like structure with thorns at the centre and periphery” [6].

The pattern of blood stains on the face of the TSM has been interpreted as evidence of him dying in a vertical position [5]. The figure ‘3’ rivulet on his forehead was attributed to “wrinkles” and a “tilting [of] the head to both sides” [4, 15]. Lavoie et al. [5] concluded from their experiments that the blood that “seem[s] to be in the hair” must have actually originated “from blood clots on the face”.

### Blood stains attributed to the *lance wound* and the *belt of blood*

The group of blood stains at the right side of the chest (at the level of the fifth or sixth rib [6] (Fig. 1)) has been related to the *lance wound* that was inflicted on the body of Jesus [4] according to the Gospel of John (“But one of the soldiers with a spear pierced his side, and forthwith came there out blood and water”, John 19:34 [14]).

Svensson and Heimburger [4] described these stains as follows: “At the right side [of the chest] a hand [sized] blood stain consisting of meandering rivulets is seen flowing in distal direction and apparently continuing around the small of the back […]. This flow emanates from a proximal, clear demarcated, oblique oval shaped wound […]. Judged from the shape and amount of blood/fluid this wound seems caused by a sharp object, able to penetrate deep into the chest cavities emptying these cavities from blood and fluid.” Faccini et al. [8] described “an oval, elongated mark (4 × 1.5 cm […]) corresponding to the area where the spear entered” as a wound that “does not show signs of vital retraction”, as well as “a bloody outflow 6 cm wide and 15 cm long”.

The meandering rivulets “continuing around the small of the back” [4] (Fig. 5d) were named the *belt of blood* by some authors [16, 17].

Many authors support the hypothesis that this group of blood stains is “of a postmortem type” with characteristics of “passive drainage” [4, 8, 15] of either a mixture of serum and blood (“blood and water”, John 19:34 [14]) from a hemothorax [3, 4] or a mixture of pleural effusion and blood from the injured heart [6, 18, 19] after opening the chest cavity with the lance postmortem. A somewhat more faded zone surrounding the *lance wound* was interpreted as a “serum ring” [4] and was therefore assumed as evidence for the hemothorax hypothesis [4].

A minority of authors—supporters of the “alive hypothesis”—interpreted the chest wound as an injury-inflicted antemortem, based in particular on the assumption that a considerable blood flow would only be possible if there was still active blood circulation [8, 20].

Borrini and Garlaschelli [17] performed “the first proper experimental bloodstain pattern analysis (BPA) on some blood stain complexes” on the TS. For the analysis of the chest wound complex, they attached a sponge soaked in synthetic blood and the size of the blood stain (6.5 × 2.5 × 3.5 cm) to the corresponding area of a mannequin torso. The mannequin held upright, and they observed vertical rivulets but no “large, filled stain as seen [on] the Shroud”. In a supine position, the rivulets from the chest wound (the sponge) did not produce a *belt of blood* pattern, as seen in the lumbar region of the TSM. In the discussion of this finding, it was concluded that “the hypothesis of a postmortem bleeding to generate the lines on the lumbar area (“belt of blood”) seems to be unrealistic”. However, these experiments have since been heavily criticised [1, 21].

### Our contribution to the discussion about the origin of the blood stains on the TS

From the point of view of a forensic pathologist, the prerequisites for obtaining sufficiently reliable conclusions about the origin of the blood stains on the TS are extremely poor. At the moment, direct access to the Shroud, to physical samples of the Shroud and even to the existing photographs is restricted or even impossible for scientists. Moreover, the only information regarding the circumstances of the unnatural death of Jesus Christ stems from the Gospels that were partly written at least 70 years after his death [22]. They report the observations of medical laypersons and, in part, even contradict each other (e.g. with regard to whether or not a washing of the body occurred after death; see discussion by Zugibe [12]).

Under these conditions, it is not possible to exactly reconstruct the origin of the blood and its transfer to the TS. Furthermore, it is certainly not possible to clarify the question of the authenticity of the TS only by assessing (the appearance of) the blood stains. Therefore, we abstain from taking part in the debates between “TS believers” and “TS sceptics”.

Nevertheless, the discussion regarding the origin of the blood stains on the TS is very intriguing—especially from a forensic scientist’s viewpoint. Since we felt that some of the published hypotheses should be investigated further, we conducted experiments that may contribute to the discussion regarding the origin of the blood stains on the TS.

The aim of our experiments was not to reconstruct the TS but to identify theoretically possible explanations for the formation of some of the stains on the TS (blood stains attributed to injuries caused by the *crown of thorns* as well as the *belt of blood* and the *serum ring* in the area of the *lance wound*).

## Material and methods

### Experiments with focus on the complex “blood stains attributed to injuries caused by the *crown of thorns*” (Fig. 2)

For each experiment, approx. 20–30 ml of blood was taken from a live proband; tubes with anticoagulant coating were used for sampling. The following experiments were performed by applying blood to the head/hair of the same person:

1. a. Simulation 1a: *Liquid/moist blood from antemortem scalp wounds is covering the hair and the face.*
  Blood was applied via syringe in dots along a circular line (corresponding to an imaginary *crown of thorns*) to the scalp as well as to the long, straight and dry hair and the frontal hairline of the proband, while the proband is sitting in an upright position. Thereafter, the proband’s head was wrapped in white cloth (100% cotton) (Fig. 2b).
  b. Simulation 1b: *Blood from antemortem scalp wounds is covering the hair and the face; blood is coagulating and/or drying; blood components are mobilised by postmortem washing.*
  After the blood from the aforementioned application (see simulation 1a) had dried, the hair was moistened, and another 20–25 ml of liquid blood was applied in the same way to the wet hair while in an upright position. Afterwards, the head was wrapped in a white cotton cloth (Fig. 2c).
2. Simulation 2: *Blood from antemortem scalp wounds is covering the hair and the face; blood is coagulating and/or drying; blood components are mobilised by postmortem washing and oiling.*
  The proband’s clean and dry hair was moistened, and approx. 10–15 ml of liquid blood was applied to the wet hair as explained in simulation 1a. The face and frontal hairline was lotioned and oiled (with commercial hair and skin products). Thereafter, 10–15 ml of liquid blood was applied in the same manner as before to the wet and oiled hair. Subsequently, the head was wrapped in white cotton cloth (Fig. 2d).

### Experiments with focus on the complex “serum ring around the *lance wound*” (Fig. 3)

**Fig. 3:**
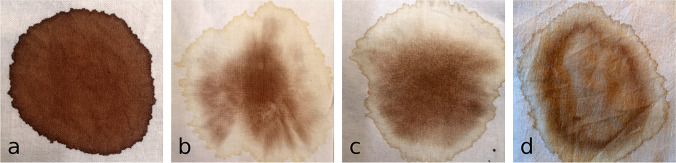
Experimental findings regarding the complex “serum ring around the *lance wound*”: dried fluid spots after application of small samples of whole blood (**a**), vital whole blood on vital serum (**b**), postmortem whole blood on postmortem serum (**c**) and samples of postmortem blood on postmortem pleural effusion (**d**) onto cotton cloths. The findings in b–d are very similar; visually, a “serum ring” can not be distinguished from a “pleural effusion ring”

Small samples of vital and postmortem blood, vital and postmortem serum and postmortem pleural effusion were applied to white cloth (100% cotton), and the spots were then dried. The postmortem samples were taken during autopsies with postmortem intervals between 1 and 13 days. The findings were photographically documented (for examples, see Fig. 3). The samples were processed as follows:

1. Vital whole blood samples, undiluted with added anticoagulants: multiple spots of 1 ml each of either vital or postmortem whole blood were applied to cotton cloths via syringe (Fig. 3a).
2. Whole blood on serum samples: multiple spots of 1 ml each of either vital or postmortem serum were applied to the cloths via syringe. Immediately thereafter, 0.5 ml of either vital (Fig. 3b) or postmortem whole blood (Fig. 3c), respectively, was added to each centre of the serum spots.
3. Whole blood on pleural effusion samples: multiple spots of 1 ml each of pleural effusion were applied to the cloths. Immediately thereafter, 0.5 ml of postmortem whole blood was added to each centre of the pleural effusion spots (Fig. 3d).

### Experiments with focus on the complex “*belt of blood*” (Fig. 4)

**Fig. 4:**
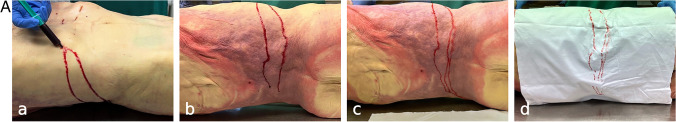
Experiments regarding the complex “*belt of blood”*. Experimental provocation of a *belt of blood*: **a** simulation of a flow of blood or bloodied water from a chest wound by application of blood to the right lateral chest wall (according to the assumed location of the *lance wound*); **b** rotation of the body to the left side; **c** tucking the cloth under the body; **d** wrapping the cloth around the body

A flow of blood or bloody water from a chest wound was simulated by applying 2 ml of either undiluted or diluted postmortem blood (dilutions 1:4 and 1:10 with saline solution) to the right lateral chest wall (at the location of the *lance wound*) of a dead body in a supine position (Fig. 4a). The body was then rotated onto the left side, so that a cotton cloth could be tucked under the back (Fig. 4b, c). Subsequently, the body was placed onto the cloth (Fig. 4d). The cloth was then carefully removed to avoid artificial blood stains.

## Results

### Experiments with focus on the complex “blood stains due to injuries caused by the *crown of thorns*” (Fig. 2)

1. a. Findings after simulation 1a *(liquid/moist blood from antemortem scalp wounds is covering the hair and the face)* (Fig. 2b):
  After applying fresh whole blood onto dry hair, the blood ran down from the frontal hairline to the forehead and face as well as the cheeks and then dried, especially at the jaw and neck. At the back of the head, the applied blood was not visible. After wrapping the head in a cloth, bloody imprints were only seen on the parts of the cloth covering the frontal/facial area of the proband. There were a few cord-like imprints and some drops corresponding to the rivulets from the frontal hairline to the forehead and cheeks. There were no blood stains found at the parts of the cloth originally covering the back of the head.
  b. Findings after simulation 1b *(blood from antemortem scalp wounds is covering the hair and the face; blood is coagulating and/or drying; blood components are mobilised by postmortem washing)* (Fig. 2c):
  After applying fresh whole blood samples onto moistened hair containing dried and coagulated residues of blood, liquid (diluted?) blood ran down fast in the same way as in experiment 1a. Especially at the jaw, the blood stains seemed more diluted. In total, more bloody imprints were visible on the cloth. The imprints mainly corresponded to the bloody rivulets from the forehead, cheeks and lower jaw. At the forehead, a few drops and some string of pearl-like imprints were found. The blood stains from the jaw seemed more blurred. Imprints from the back of the head were washed out.
2. Findings after simulation 2 *(blood from antemortem scalp wounds is covering the hair and the face; blood is coagulating and/or drying; blood components are mobilised by postmortem washing and oiling)* (Fig. 2d):
  After applying blood onto moistened hair, oiling the skin and frontal hairline and applying fresh whole blood samples, the blood ran down slowly and dried more evenly distributed on the face and cheeks. There were a few string of pearl-like stains on the parts of the cloth covering the frontal area; the imprints on the dorsal part of the cloth seemed a bit washed out and tangled in the centre of the contact surface. These findings were most similar to the stains seen on the TS (Fig. 2a).

### Experiments with focus on the complex “serum ring around the *lance wound*” (Fig. 3)

The application of the different bodily fluid samples to cotton cloth revealed the following findings:

1. Undiluted whole blood samples:

Strongly coloured stains with well-defined borders without a surrounding “ring” or margin (Fig. 3a), no visible differences between vital and postmortem blood.

2. “Whole blood on serum” samples:

Less coloured “rings” around a darker-stained centre (Fig. 3b, c). Identical findings for postmortem and vital blood samples.

3. “Whole blood on pleural effusion” samples:

Less coloured “rings” around a darker-stained and inhomogeneously coloured centre (Fig. 3d).

### Experiments with focus on the complex ‘*belt of blood*’

**Fig. 5:**
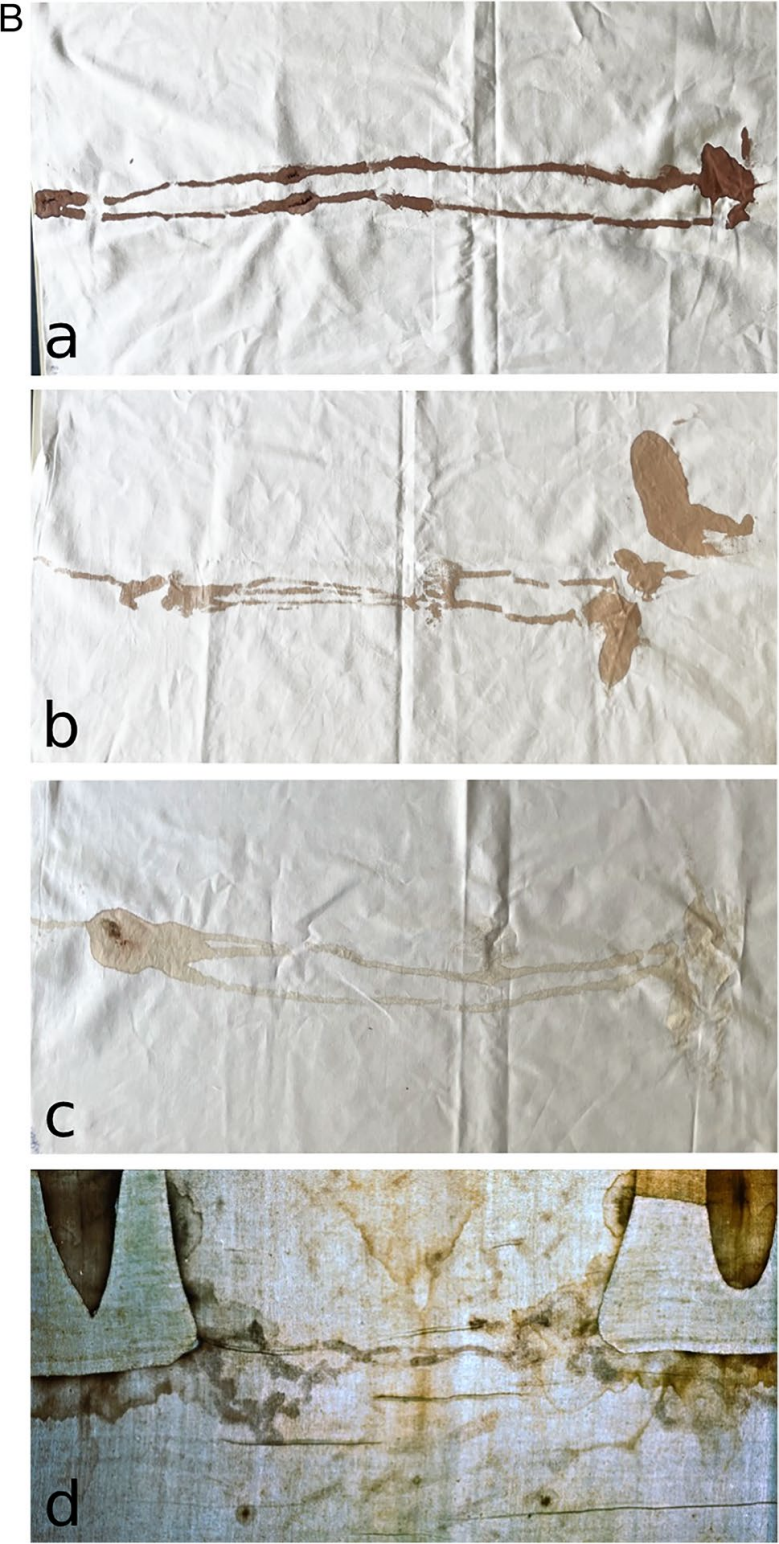
Experiments regarding the complex “*belt of blood”*. Experimental findings on the cloth after the provocation of a *belt of blood* as illustrated in Fig. 4: **a** using undiluted blood and **b**, **c** using watery dilutions of blood (1:4 and 1:10, respectively); the findings appear very similar to the *belt of blood* on the TS **(d)** (*photo from *www.shroudphotos.com*, ©Vernon Miller, 1978*), especially when diluted blood samples were used

As depicted in Fig. 4a–c, a rotation of the body to the left in order to tuck the cloth under the back (Fig. 4d) resulted in blood rivulets running down the back approximately perpendicular to the longitudinal axis of the body (Fig. 5a–c). These rivulets become finer and more branched with higher dilutions of the blood. At the side opposite of the *lance wound*, the rivulets converge to a circumscribed but more widespread area. Especially using diluted blood, the findings of the experiments appear very similar to the *belt of blood* on the TS (Fig. 5c, d).

## Discussion

In many respects, the discussion of our findings relates to the information given by the gospels and insofar hypothetically assumes that the TS is the burial cloth of a man (TSM) who underwent the same tortures as Jesus of Nazareth.

To approach the questions regarding the origin of the blood stains on the TS, one has to take into account the possible states of the body of the TSM in the course of the events reported.

The acts of violence described by the gospels (scourging, wearing a *crown of thorns*, punching, crucifixion) would result in multiple bleeding injuries; the body of the wounded man would already be covered in blood after his way to Golgotha and even more so after crucifixion. Depending on the pathophysiological effects of the injuries and on external influences, parts of the blood would soon begin to clot in, on and/or around the wounds and blood stains; smears and rivulets on the skin would dry. At the very latest, all active bleeding from skin wounds would cease at the time of death.

Bevilacqua et al. [23] presented a “probable thanato-chronology” of the TSM. According to their assumptions, the TSM died on Friday at 3.00 p.m.; the removal of the body from the cross is assigned to Friday 5:00 p.m., the “transport and preparation of the corpse on the sepulchral stone” as well as the “wrapping of the body in the TS” to Friday 5:30 p.m. (2.5 h after death), the “closing [of] the entrance of the sepulchre” to Friday 6:00 p.m. and the “resurrection” to Sunday, c. 3:00–5:00 a.m.

Presumably, the blood on the body of the TSM had mostly dried by the time of his removal from the cross (2.5 h after death, in a hot and dry environment). If a blood-covered body in this condition was wrapped in linen, there would be no blood transfer at all or, at the most, a very sparse amount of blood transfer from regions where the blood had not yet completely dried or where dried blood was chipped or rubbed off, resulting in no or only discrete and irregular blood stains (smudges or flakes of dried blood) on the linen. Under these conditions, it is difficult to imagine the formation of such well-defined blood stains as observed on the TS. This assumption is in line with the results of the experiments of Lavoie et al. [11].

Thus, the blood (or the blood components) must have been mostly liquid/moist at the time of their transfer from the body to the linen. Accordingly, at least three hypotheses on the theoretical remobilisation of primarily dried blood (clots) have so far been presented in the literature:

I. A postmortem external fibrinolytic (“melting”) activity enabled a postmortem transfer of (primarily coagulated) blood to the linen [4, 8–10].
II. A postmortem smudging of blood from clots in a moist environment created the conditions necessary for a transfer [4, 6, 11].
III. The transfer of blood (or blood components) with the result of a precise and undisturbed blood stain pattern is due to a secondary mobilisation of blood by washing the body before placing it onto the burial cloth [12].

The “postmortem fibrinolysis hypothesis” (I.) as well as the “postmortem smudging hypothesis” (II.) is obviously based on the assumption that due to influences of the (humid?) environment in the sepulchre, (over a period of c. 9.5–11.5 h according to Bevilacqua et al. [23]) already coagulated and/or dried blood (components) became liquid/moist and thus transferable. Both hypotheses focus on blood clots. Lavoie et al. [11], however, emphasise the differentiation between blood stains from “clots” (at the head and arms) and those from the “scourge marks and the blood flow on the dorsal image” because of the “different category regarding their formation and moisture content”; how these “different categories” would influence a blood transfer remains unclear. Moreover, these hypotheses lack explanations for the very well-defined shapes and similar appearance of blood stains at the front and the back (exposed to different environmental conditions, especially with regard to humidity).

Hypothesis III (mobilisation of blood by washing the body before wrapping it into the TS) concerns the highly controversial discussion whether the TSM was washed prior to being wrapped in the shroud or not. This question shall not be the focus of this paper and is beyond our professional competence. From a forensic pathologist’s point of view, hypothesis III appears very interesting, since a postmortem dispersion of blood (or blood components in water) can often be observed in the autopsy room after postmortem washing/cleaning of open wounds or blood-soaked hair. Zugibe [12] observed “reasonable good impressions of the wounds” on linen and paper towels gently touched against wounds that had been washed postmortem. Furthermore, he described small rivulets of “blood” (more likely bloody water) that appeared after wounds were briefly washed postmortem. The results of the experiments performed by Zugibe [12] appear highly plausible from a forensic pathologist’s viewpoint. Since Zugibe [12] reports only few experiments, we further investigated the question of whether a transfer of blood (or blood components) can be facilitated by washing the body postmortem.

In our experiments, we were able to produce blood stains that were very similar to the findings on the TS under certain conditions.

The experiments with focus on the blood stains due to injuries caused by the *crown of thorns* showed that by simulating the sequence “*blood from antemortem scalp wounds is covering hair and face – blood is coagulating and/or drying – blood components are mobilised by postmortem washing (with and without oiling)”*, blood (or blood components) was mobilised and could be transferred to a cloth wrapped around the head. By applying oil in addition to washing, findings most similar to the stains seen on the TS could be provoked (Fig. 2d). This result is especially interesting in light of the Gospel of John: “Then took they the body of Jesus, and wound it in linen clothes with the spices, as the manner of the Jews is to bury” (John 19:40 [14]).

A blood stain pattern very similar to the *belt of blood* on the TS was successfully recreated by simulating the following possible sequence of events. The body was lying in a supine position in the sepulchre (and was possibly washed). To put the body onto the shroud, the body was rotated to the left side so the shroud could be tucked under the back (as depicted in Fig. 4). In our experiments, this rotation of the body resulted in a postmortem flow of blood or bloody fluid across the back and, after transfer to the cloth, in similar rivulets to those of the *belt of blood* on the TS (Fig. 5). A fine branching of the rivulets as seen on the TS could be provoked with a watery dilution of blood. This finding may be interpreted as another indication for a postmortem washing of the body. During the washing process, water may have entered the *lance wound*, may have mixed with blood in/on/around the wound and may have been mobilised by the rotation of the body.

The only blood stain that covers a more extensive area (“hand size” [4]) is the blood stain in the location of the *lance wound* which, according to the gospels, was inflicted postmortem. Lavoie et al. [13] emphasise that according to Jewish burial customs, a body was not washed if “death is by violence and blood flows at the time of death”. According to the gospels, witnesses observed the passion and death of Jesus Christ and should have seen which wounds were inflicted antemortem and which postmortem. It would be an interesting question to ask experts of Jewish burial customs whether it is conceivable that with the knowledge of witnesses being present, only the area of the postmortem wound was left out during washing, while the rest of the body was washed.

Another special feature of the blood stains in the area of the *lance wound* is a surrounding margin that has been interpreted as a “serum ring” [4]. We were able to reproduce very similar findings (blood stains with surrounding less coloured margins or “rings”) by firstly applying small samples of either vital or postmortem serum or postmortem pleural effusion (all relatively clear fluids) onto cloth and secondly small samples of either vital or postmortem whole blood, respectively, on top of the clear fluid spots. For both the “whole blood on serum” and “whole blood on pleural effusion” samples, the findings were very similar. A differentiation between a “serum ring” and a “pleural effusion ring” surrounding the blood stain from the *lance wound* on the photos of the TS seems to be impossible; thus, the so-called “serum ring” may also very well be a “pleural effusion ring”. Our findings cannot contribute to the discussion of the “hemothorax hypothesis” [4, 8] versus the “pleural effusion hypothesis” as an explanation for the flow of “blood and water” as described in the gospels (John 19:34 [14]).

## Conclusions

It is obvious that any attempt to interpret the “blood stain pattern” on the TS has serious limitations. At the moment, any interpretation can only be based on photographs. And even if one was to assume that the TS is indeed the burial cloth of Jesus of Nazareth and that the relevant stains are, in fact, blood stains, there is little scientifically verified information concerning (for the interpretation of the “blood stain pattern”) relevant details of what exactly was done with or what happened to the cloth around 2000 years ago and ever since. We agree with Jumper et al. [7] who stated that “science is really not in a position to ever prove categorically that the Shroud is authentic” (i.e. Jesus’ burial cloth).

Nevertheless, it seems remarkable that we were able to reproduce findings similar to the blood stains on the TS. The results of our experiments do not, however, indicate that the blood stains on the TS have formed exactly as we were able to provoke them; they only open up new lines of discussion about possible explanations (amongst others?) for the formation of blood stains on the TS.

## Data Availability

Data sharing is not applicable to this article as no datasets were generated or analysed during the current study.
